# Optical Properties of Nitrogen-Substituted Strontium Titanate Thin Films Prepared by Pulsed Laser Deposition

**DOI:** 10.3390/ma2031388

**Published:** 2009-09-22

**Authors:** Ivan Marozau, Andrey Shkabko, Max Döbeli, Thomas Lippert, Dimitri Logvinovich, Marc Mallepell, Christof W. Schneider, Anke Weidenkaff, Alexander Wokaun

**Affiliations:** 1 Paul Scherrer Institut, CH-5232 Villigen PSI, Switzerland; E-Mails: ivan.marozau@psi.ch (I.M.); christof.schneider@psi.ch (C.W.S.); alexander.wokaun@psi.ch (A.W.); 2 Empa, Überlandstrasse 129, CH-8600 Dübendorf, Switzerland; E-Mails: Andrey.Shkabko@empa.ch (A.S.); Dimitri.Logvinovich@empa.ch (D.L.); Anke.Weidenkaff@empa.ch (A.W.); 3 Ion Beam Physics, Paul Scherrer Institut and ETH Zurich, CH-8093 Zurich, Switzerland; E-Mails: doebeli@phys.ethz.ch (M.D.); mallepell@phys.ethz.ch (M.M.)

**Keywords:** oxynitrides, strontium titanate, pulsed laser deposition, thin films, optical properties

## Abstract

Perovskite-type N‑substituted SrTiO_3_ thin films with a preferential (001) orientation were grown by pulsed laser deposition on (001)-oriented MgO and LaAlO_3_ substrates. Application of N_2_ or ammonia using a synchronized reactive gas pulse produces SrTiO_3-x_:N_x_ films with a nitrogen content of up to 4.1 at.% if prepared with the NH_3_ gas pulse at a substrate temperature of 720 °C. Incorporating nitrogen in SrTiO_3_ results in an optical absorption at 370‑460 nm associated with localized N(2p) orbitals. The estimated energy of these levels is ≈2.7 eV below the conduction band. In addition, the optical absorption increases gradually with increasing nitrogen content.

## 1. Introduction

Perovskite-type oxides with an *ABO_3_* structure are attractive due to the large variation of their properties. These materials are widely used for applications in microelectronics or catalysis [1]. One possible way to change these materials’ properties and to adjust them to specific applications is to change their chemical composition. Typically, the cationic stoichiometry in the *A* and *B* sublattices of the crystal structure is changed [2,3]. The second possibility, an anionic substitution, is a much less explored approach, no doubt due to the difficult synthesis of these materials [4]. However, this type of substitution is considered to be a very promising approach to modify various properties of perovskites [4,5,6,7,8,9,10,11,12].

The study presented herein was focused on the one‑step preparation of nitrogen-substituted perovskite-type strontium titanate (SrTiO_3_:N) thin films by pulsed laser deposition (PLD) and to investigate of the influence of the incorporated nitrogen on the thin films’ optical properties. It has been reported that the incorporation of small amounts of nitrogen (0.06‑0.26 at.%) into the lattice of SrTiO_3_ or La‑doped SrTiO_3_ powders creates populated localized N(2*p*) levels inside the band gap of SrTiO_3_ [13]. This change in the electronic structure results in visible light absorption, which is of interest for photochemical applications under visible light irradiation [13]. In a previous paper we have studied optical properties of SrTiO_3_:N thin films with nitrogen contents of up to 2.3 at.%, which were deposited onto MgO substrates [14]. The aim of this study was to prepare films with a higher nitrogen contents than previously reported and to investigate their optical properties as a function of the nitrogen content.

## 2. Experimental

SrTiO_3_:N thin films were prepared by PLD and a modification of conventional PLD, termed pulsed reactive crossed beam laser ablation (PRCLA). The latter deposition technique provides a better control over the anionic stoichiometry during ablation [15,16]. A specific feature of this technique is the utilization of a pulsed gas injection (gas pulse), synchronized with the laser pulse. The injected gas crosses the ablation plume close to its origin resulting in dissociation and activation of the gas pulse molecules via collisions with the high-energetic ablation plasma species. The utilization of oxygen-containing gases helps to overcome oxygen deficiencies in films [15], while the use of nitrogen (N_2_) or ammonia (NH_3_) allows the deposition of nitrogen-substituted films [14,17]. In this study, both nitridizing gases were used for the gas pulse and background gas. The background gas pressure was fixed at 8 × 10^‑4^ mbar, whereas the total deposition pressure was between (1‑2) × 10^‑3^ mbar. The ablation of SrTiO_3_ took place from a sintered, cylindrical ceramic target. Reference SrTiO_3‑x_ thin films without incorporated nitrogen were deposited by standard PLD in vacuum (*P* = 1 × 10^‑5^ mbar). One SrTiO_3‑x_ film has also been grown by conventional PLD with an NH_3_ background gas (*P* = 1 × 10^‑3^ mbar) to compare the efficiency of both deposition techniques with respect to the nitrogen incorporation into the as-grown films.

Films with thicknesses of 500 ± 50 nm were deposited with a KrF excimer laser (λ = 248 nm, pulse duration 20 ns) at a laser fluence of 5.5 ± 0.5 J·cm^-2^. The distance between the ceramic SrTiO_3_ target and the substrate was 5.0 ± 0.1 cm. Two different (001)‑oriented substrates were used: MgO (*a* = 4.216 Å) and LaAlO_3_ (LAO, *a* = 3.790 Å). The optimized substrate temperature (*T*_S_) for the film deposition was 650 ± 25 °C. Several films were also grown at *T*_S_ between 570 and 720 °C to study the influence of the substrate temperature on the film composition and the respective optical properties.

The chemical film composition (Sr:Ti:O concentration ratio) was determined by Rutherford backscattering (RBS) [18] with an experimental uncertainty of typically ±3% for cations (Sr and Ti) and ±5% for oxygen. The nitrogen to oxygen ratio for the investigated films was calculated from a combined RBS and elastic recoil detection analysis (ERDA) approach with a relative experimental uncertainty of 3% (see table 1) [19]. This corresponds to an uncertainty of 0.12 at.% for the film with the maximum N content of 4.1 at.%.

Grazing incidence *X*‑ray diffraction measurements on selected samples were performed on a Phillips X’Pert diffractometer (Cu K_α_ irradiation, incident angle of 1°, 2Θ range of 20‑80°, step width 0.05°, 0.5 s/step) to verify the phase composition of the deposited SrTiO_3_:N films. The film texture was studied using a Siemens D5000 diffractometer (Cu K_α_ irradiation, 2Θ range of 20‑80°, step width 0.005°, 0.3 s/step) in the Θ‑2Θ mode along the *c*‑axis of the substrate.

The transmittance, *T*, of these films was measured by a Cary 500 Scan UV‑Vis‑NIR spectrophotometer in the wavelength (*λ*) range of 190‑2000 nm (6.5‑0.6 eV). The measurements were performed in a two‑beam configuration using a blank substrate as reference. The optical band gap energy (*E_g_*) and energy level positions inside the band gap (*E_N_*) were determined by linear extrapolation of *(A·hυ)^2^* vs. the photon energy in the appropriate linear region using the Tauc equation for direct electronic transitions [20]:

(*A* · *h*υ)^2^ ~ (*h*υ − *E*) (1)

where *hυ* is the photon energy; *E* is the optical band gap energy or energy levels inside the band gap; *A* is the film absorbance estimated from the film transmittance as:

*A* = −lg(*T*) (2)

## 3. Results and Discussion

### 3.1. Crystal structure of SrTiO_3_:N thin films

**Figure 1:**
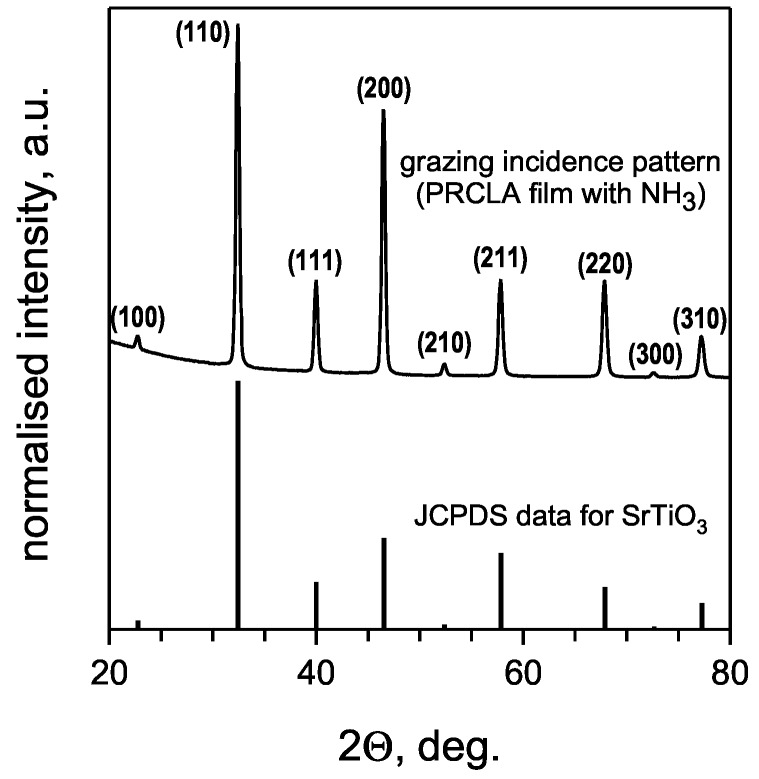
Grazing incidence diffraction pattern of SrTiO_3_:N. All observed reflexes are in agreement with the reference data for SrTiO_3_, confirming the perovskite-type phase purity.

**Figure 2:**
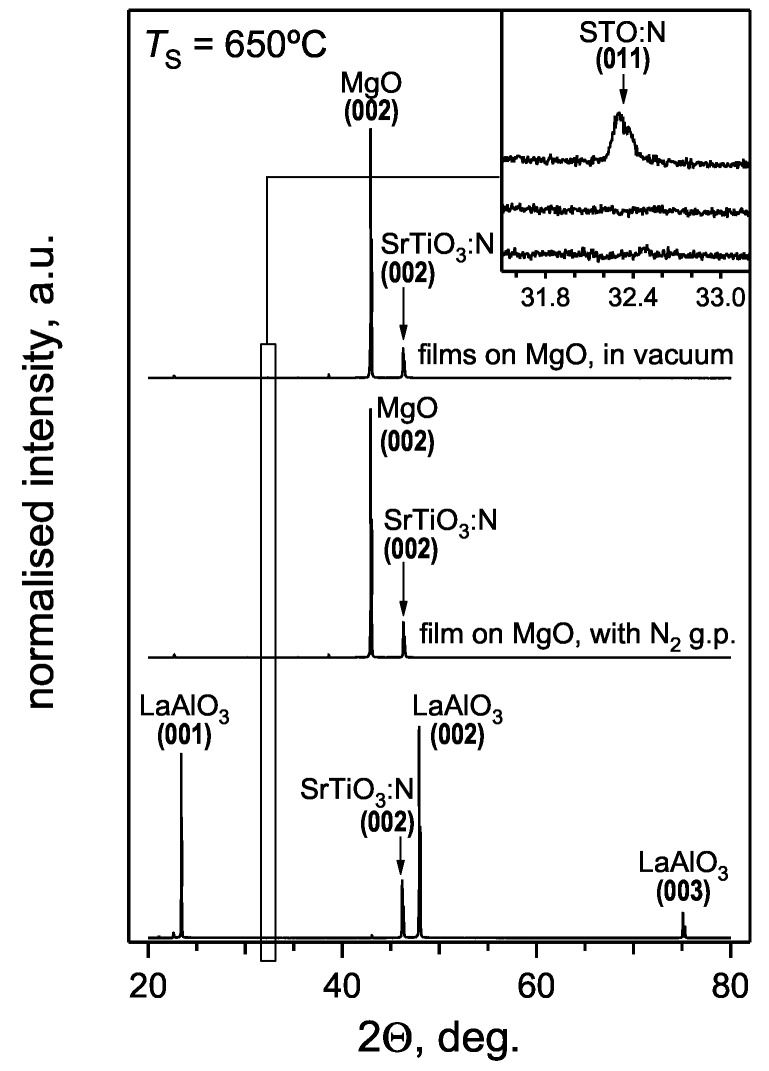
Θ‑2Θ diffraction patterns along the *c*-axis of films grown on (001)-oriented MgO and LaAlO_3_ substrates. The insert shows the detailed region around the (011) film reflection. Films grown on LaAlO_3_ exhibit a single (001) out-of-plain orientation, while some films deposited on MgO reveal a mixed-oriented growth.

Grazing incidence XRD analysis of the deposited films revealed the formation of a pure perovskite-type phase. All observed film reflections are in good agreement with the SrTiO_3_ diffraction pattern from the JCPDS database (Figure 1). XRD in Θ‑2Θ mode revealed a single (001) out-of-plane orientation for SrTiO_3_:N films grown on (001) LaAlO_3_ (Figure 2). Some of the (001) oriented films deposited on (001) MgO exhibit in addition a weak (011) contribution (insert in Figure 2). This indicates a non-perfect out-of-plane orientation of the SrTiO_3_:N films grown on MgO which is likely to be related to the relatively large lattice mismatch of about +7.6%, compared to -2.9% for the LaAlO_3_ substrates.

The differences in the crystallinity of the SrTiO_3_:N films on different substrates have no pronounced influence on the optical properties of the films. The data analysis and discussion in this paper is focused on the films deposited on LaAlO_3_ substrates, although exactly the same phenomena were observed for the films grown on MgO. All films have a typical thickness of 500 ± 50 nm, allowing a direct comparison of their transmittance spectra.

### 3.2. Chemical composition of SrTiO_3_:N thin films

Differences in the chemical compositions of films using the same deposition conditions but on different substrates lie within the statistical deviation. Thus, no pronounced influence of the used substrate on the chemical composition of the films was found. The elemental composition depends mainly on the deposition medium, i.e., which gas was used for the gas pulse. To analyze the film composition, all studied samples are grouped in three series: films grown in vacuum, with the N_2_ gas pulse, and with the NH_3_ gas pulse. The average compositions for these film series are presented in Table 1. The stoichiometry factors for Sr, Ti and O were calculated assuming the following normalization of the film composition: Sr_1-z_Ti_1+z_O_3-x_N_y_, where *z* represents the deviation from the ideal cationic stoichiometry Sr:Ti = 1:1, *x* is the oxygen deficiency with respect to the ideal stoichiometry factor of 3, and *y* is the stoichiometry factor of nitrogen in the films. The amount of incorporated nitrogen is also often expressed by the relative content of nitrogen atoms, [N] (in atomic %):

(3) $${Sr}_{1-z}{Ti}_{1+z}O_{3-x}N_{y}:\;[N]=\frac{y}{5-x+y}$$

It is noteworthy that the Sr content in these films (1-*z*) is consistently lower than the Ti content (1+*z*), which is not yet fully understood. This effect is more pronounced for films deposited under vacuum and with the NH_3_ gas pulse. This effect has also been observed for SrTiO_3_ films grown by conventional PLD [21], during the surface treatment of SrTiO_3_ single crystals in an ammonia microwave-induced plasma [22] and during ion sputtering of a SrTiO_3_ surfaces [23].

**Table 1:** Properties of the N‑substituted SrTiO_3_ films grown under different deposition conditions at 650 °C.

Deposition Conditions	Average Film Composition	*E*_g_ ± 0.05 [eV]	*E*_N_ ± 0.10 [eV]
conventional PLD, NH_3_ background	Sr_0.98_Ti_1.02_O_2.9_N_0.032_		
PRCLA, under vacuum	Sr_0.97_Ti_1.03_O_2.52_	3.36	
PRCLA, with N_2_ gas pulse	Sr_0.98_Ti_1.02_O_2.89_N_0.049_	3.38	2.70
PRCLA, with NH_3_ gas pulse	Sr_0.95_Ti_1.05_O_2.73_N_0.112_	3.38	2.68

**Figure 3:**
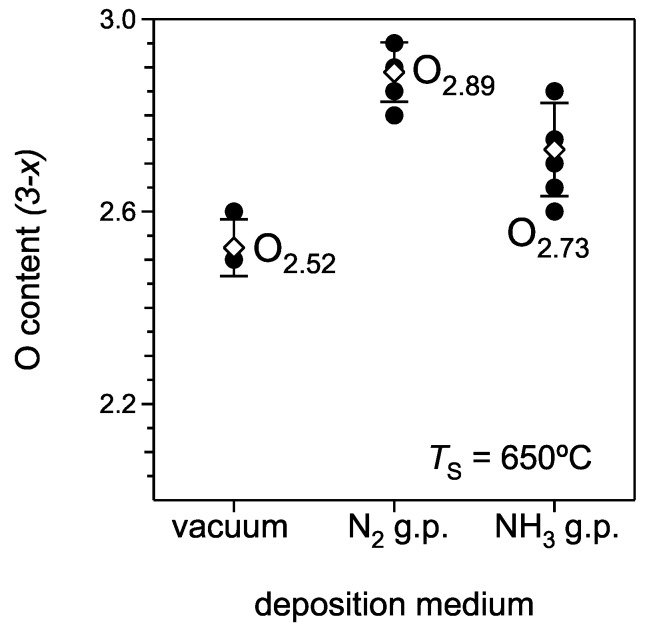
Average oxygen stoichiometry factors of films prepared in vacuum, with the ammonia and the nitrogen gas pulse. The *open symbols* are the calculated mean values and the *error bars* show the respective confidence intervals for the different deposition runs.

Figure 3 shows the oxygen content for different deposition conditions. The oxygen content calculated from the RBS data is smaller than the ideal stoichiometric value of 3 for the three-dimensional perovskite-type structure. Oxygen deficiencies in films are a typical issue for oxide materials grown by PLD [15,24]. Light elements, such as oxygen, are almost always deficient in a film due to a larger degree of scattering in the plasma plume and a lower sticking coefficient on the heated substrate as compared to heavier metallic species. Different sources of oxygen (e.g., background gas, gas pulse, or RF-induced plasma) are used for the deposition of perovskite-type oxide films by PLD in order to improve the oxygen content in the films [15,24,25,26]. However, in the present study none of these oxygen sources can be used to improve the oxygen content in N‑substituted SrTiO_3_ films because a simultaneous use of oxygen- and nitrogen-containing sources yields N‑free SrTiO_3_ films [17]. This is due to the lower thermodynamic stability of the oxynitride phase with respect to the pure oxide phase, which leads to the lower affinity of the growing film to the nitrogen species compared to oxygen species when both are present in the plasma plume.

The oxygen stoichiometry in the investigated films is different, although all three films series were deposited in oxygen-free media (vacuum, N_2_ and NH_3_ gas pulse). The lowest oxygen content is observed in reference films deposited at 1 × 10^‑5^ mbar (vacuum). The obtained average oxygen stoichiometry factor of 2.52 ± 0.06 is quite similar to the value of 2.50 for the SrTiO_3‑x_ film grown at a pressure of about 1 × 10^‑7^ mbar [27]. Films deposited by PRCLA with a N_2_ or NH_3_ gas pulse yields films with higher oxygen contents (Figure 3). This can be attributed to two effects:

1. Plasma species in a laser plume have a much higher kinetic energy with a deposition in vacuum causing re-sputtering of light elements, such as O, from the surface of the growing film. During the deposition with the gas pulse, the ablated species are slowed down considerably and are not energetic enough to cause a significant re-sputtering [28,29,30]. As a result the oxygen content in a film becomes higher.
2. A minor O_2_ impurity in the used gases (~0.01%) and the very high affinity of the growing film to oxygen species become important for the final oxygen content in films. The average oxygen stoichiometry factors for films deposited with N_2_ and NH_3_ gas pulses are 2.89 ± 0.06 and 2.73 ± 0.10, respectively.

A deposition with ammonia results in a lower oxygen content, probably due to the reducing properties of this gas. Here, hydrogen-containing species from the NH_3_ gas pulse can react with oxygen species and hence reduce the amount of active oxygen. However, the oxygen content in these films is still higher than in the reference films deposited in vacuum.

Elastic recoil detection analysis confirms the absence of nitrogen in the reference films deposited in vacuum, while a deposition by conventional PLD or PRCLA yields films with incorporated nitrogen (Table 1). A comparison of these two techniques shows that the amount of nitrogen in these films is a factor of 3.5 higher for PRCLA (2.31 at.%) when compared to conventional PLD (0.65 at.%) if the same nitridizing source (NH_3_) is used. This result shows clearly the importance and advantages of using the synchronized reactive gas expansion in PRCLA to control the anionic composition of the film. An activation of the injected gas pulse molecules via collisions with the highly energetic ablation plasma species in the used experimental configuration yields very reactive N‑containing species. This is a necessary boundary condition to have N incorporated into the growing film.

**Figure 4:**
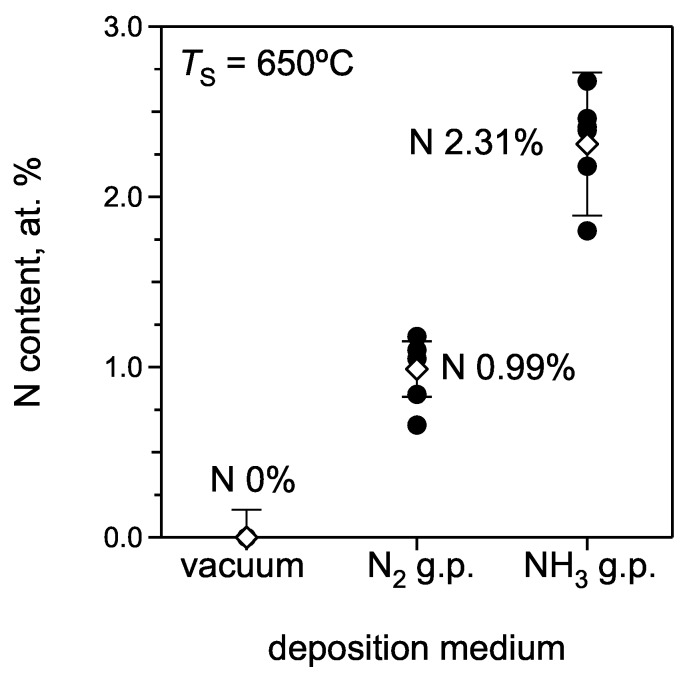
Average nitrogen content of films prepared in vacuum, with the ammonia and the nitrogen gas pulse. The *open symbols* are the calculated mean values and the *error bars* show the respective confidence intervals for the different deposition runs.

The relative nitrogen content in the films grown by PRCLA with different nitridizing sources is shown in Figure 4. The average nitrogen content in films deposited with the N_2_ gas pulse is 0.99 ± 0.16 at.% while for the NH_3_ gas pulse it is 2.31 ± 0.42 at.%. The pronounced difference in the nitrogen content between the samples grown with nitrogen and ammonia gas pulses can be explained as follows:

1. Different dissociation energy of N_2_ and NH_3_ molecules. Active atomic nitrogen is probably the most important species for the formation of oxynitrides [31]. During the deposition of thin oxynitride films by PLD these species are mainly produced by the dissociation of the gas pulse and background gas molecules via collisions with the high energetic ablated species from the target. The N_2_ molecule is thermodynamically very stable and has a dissociation energy of 945 kJ·mol^‑1^ (~9.8 eV) [32], which is considerably higher compared to the average dissociation energy for one N‑H bond in an NH_3_ molecule of 391 kJ·mol^‑1^ (~4.1 eV) [32]. Typical ion energies in PRCLA (close to the target) vary in the range of 5‑15 eV [33]. Thus, it is possible to disproportionate both nitrogen and ammonia molecules and produce active N‑containing species in the PRCLA process via collisions of the ablation plume species with the gas pulse molecules. However, smaller chemical bond energies and the possibility of a consecutive detachment of hydrogen atoms in NH_3_ makes this process more likely if compared to N_2_. This results in the higher concentration of atomic N species in the plasma, and in a larger nitrogen content in films grown with the NH_3_ gas pulse.
2. Reducing properties of ammonia and related reaction products. As already pointed out, they can capture the oxygen species in the plasma and at the surface of the growing film, thereby reducing the oxygen content in films. This enhances the number of vacant anionic sites in the crystal lattice available for nitrogen incorporation.

**Figure 5:**
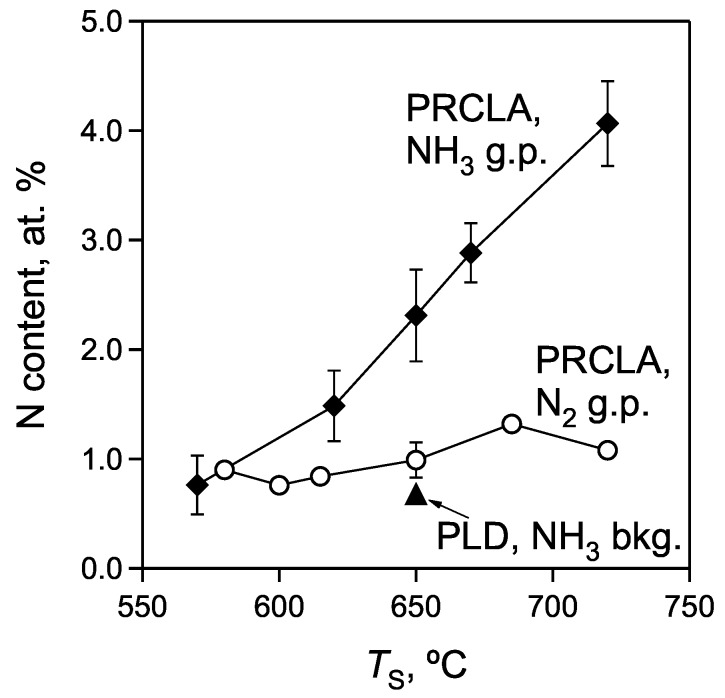
Substrate temperature dependence of the nitrogen content in SrTiO_3_:N films grown by PRCLA with the different gas pulses and by conventional PLD with an ammonia background.

The influence of the substrate temperature (*T*_S_) on the nitrogen content in SrTiO_3_:N films was also studied in more detail. The variation of the nitrogen content vs. *T*_S_ in the films grown with the N_2_ gas pulse is presented in Figure 5. The N concentration exhibits no obvious dependence on the substrate temperature. It increases slightly with increasing *T*_S_ in the range from 580‑720 °C, probably due to an enhanced kinetics for the N incorporation [17]. Deposition with the ammonia gas pulse reveals a completely different influence of the substrate temperature (Figure 5). The nitrogen content increases with increasing *T*_S_ in the studied temperature range of 570‑720 °C. The probable reason for the observed difference in temperature dependence for the N_2_ and NH_3_ gas pulse is the different thermal stability of these molecules. N_2_ is thermally stable in vacuum at temperatures up to 720 °C, while NH_3_ molecules can dissociate at the heated substrate surface, producing different N- and H-containing species, such as N, NH, NH_2_, H, N_2_, H_2_. The presence of additional atomic nitrogen combined with the reducing properties of H-containing species can result in additional nitrogen incorporation, yielding the larger final N content in the film. The dissociation velocity of ammonia is larger at higher temperatures, resulting in a higher degree of dissociation and therefore a higher nitrogen content in the films which reaches values of up to 4.06 ± 0.39 at.% at *T*_S_ of 720 °C. This value is considerably larger than the nitrogen content in SrTiO_3_:N reported elsewhere [13] and only limited by the heater design used in this study. A higher N-content of up to 8.4% can be realized at higher temperatures and different preparation conditions [22,34]. 

### 3.3. Optical properties of SrTiO_3_:N thin films

**Figure 6:**
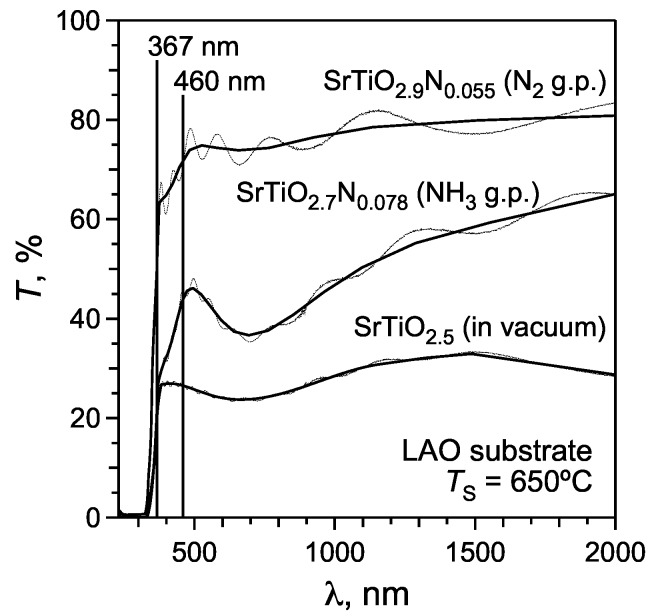
Optical transmittance spectra of the films grown on LaAlO_3_ substrates at *T*_S_ of 650 °C.

Figure 6 shows the UV‑vis‑NIR transmission spectra of SrTiO_3_:N films deposited on LAO substrates in different deposition media. As‑acquired spectra (*thin lines* on the graph) reveal typical interference fringes resulting from the interference of the light reflected from the film surface and film-substrate interface. For better visualization of the spectra the “average” curves were plotted through the half‑maxima of the interference fringes (*thick solid lines* in Figure 6). The transmittance spectra exhibit three different regions where light absorption occurs through different mechanisms:

1. Absorption of IR and visible light in a wavelength range of 460‑2,000 nm, which corresponds to photon energies of ~0.6‑2.7 eV. Absorption of these low‑energetic photons is attributed to the electronic transitions within the conduction band of reduced SrTiO_3_ [35]. Therefore, films with larger anionic deficiencies (i.e., with higher Ti^3+^ contents) reveal stronger absorption and lower transmittance (*T*) in this wavelength region, i.e., *T* (film in vacuum) < *T* (film with NH_3_ gas pulse) < *T* (film with N_2_ gas pulse).
2. A broad absorption band at wavelengths below 367 nm (3.38 eV) is attributed to the band gap of SrTiO_3_ and occurs through excitation of the valence band electrons to the conduction band. The large electron density in the valence band results in an almost complete absorption of UV light in this wavelength region.
3. The absorption shoulder between 367 and 460 nm is a specific feature of N‑substituted SrTiO_3_ which is not observed in stoichiometric or reduced strontium titanate [13]. This absorption shoulder is attributed to the electron transitions from the localized populated N(2*p*) states to the conduction band.

In 3*d*^0^ perovskites, such as SrTiO_3_, the top of the conduction band is formed by the O(2*p*) orbitals [20,36]. The energy diagram is changed when N atoms substitute O in the perovskite-type structure. The energy of the N(2*p*) orbitals is higher compared to oxygen. The populated N(2*p*) levels are therefore located inside the band gap, close to the top of the valence band in the energy diagram of SrTiO_3_. It has been shown, that for small nitrogen concentrations of ~0.06‑0.26 at.% in SrTiO_3_ the N levels inside the band gap are isolated [13], whereas for higher nitrogen concentrations (e.g., 20 at.% in LaTiO_2_N) the N orbitals “shift up” the top of the conduction band, therefore decreasing the band gap energy [5,8].

The absorption spectra of the studied SrTiO_3_:N films reveal only an absorption shoulder at 367‑460 nm due to the nitrogen incorporation, but not a complete absorption of the light, which is characteristic for the reduction of the band gap. This suggests that nitrogen forms separated N(2*p*) energy levels inside the band gap, which is similar to previously published data [13]. It is noteworthy that SrTiO_3_:N films with a larger N content (grown with the NH_3_ gas pulse) reveal stronger absorption associated with the N(2*p*) states when compared to the films grown with the N_2_ gas pulse, where the nitrogen content is smaller (Figure 6). This confirms also the above suggested assumption of separated N(2*p*) levels.

**Figure 7:**
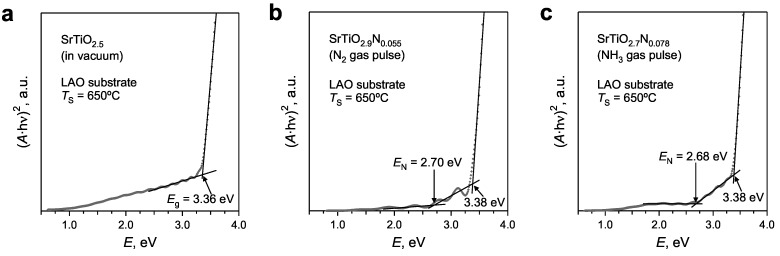
Tauc plots derived from the transmittance spectra of SrTiO_3_:N films deposited **(a)** in vacuum, **(b)** with the nitrogen, and **(c)** with the ammonia gas pulses.

The band gap energy (*E*_g_) and energy of the localized N(2*p*) levels (*E*_N_) with respect to the Fermi level are calculated from the Tauc plots (Figure 7). Reduced SrTiO_3-x_ reference films without incorporated nitrogen reveal only one slope corresponding to the band gap energy of ~3.36 eV (Figure 7a), whereas N‑substituted films exhibit an additional slope, corresponding to the N(2*p*) levels with an energy, *E*_N_, of ~2.70 eV (Figure 7b and Figure 7c). The calculated band gap energy of 3.38 ± 0.05 eV is essentially the same for all studied films (Table 1). This value is larger than the band gap energy in the stoichiometric bulk SrTiO_3_ (*E*_g_ = 3.20 eV, [37]). The difference is most probably related to the different composition of the films, i.e., anionic deficiency and/or the small Sr understoichiometry. The energy of the N(2*p*) levels in SrTiO_3_:N films *E*_N_ = 2.70 ± 0.10 eV is similar to the 2.8 eV reported in the literature for SrTiO_3_:N powders [13]. The formation of the localized N(2*p*) states inside the band gap of N‑substituted SrTiO_3_ results in the absorption of visible light photons and, therefore, suggests a potential application of these materials for photochemical applications. It has been shown previously [13] that a photocatalytic oxidation of gaseous 2‑propanol to acetone by SrTiO_3_:N powders is possible under visible light irradiation. The incorporation of a larger amount of nitrogen and inhibiting the formation of Ti^3+^ in N‑substituted SrTiO_3_ would improve the potential of the oxynitrides for photocatalytic applications [13,38].

**Figure 8:**
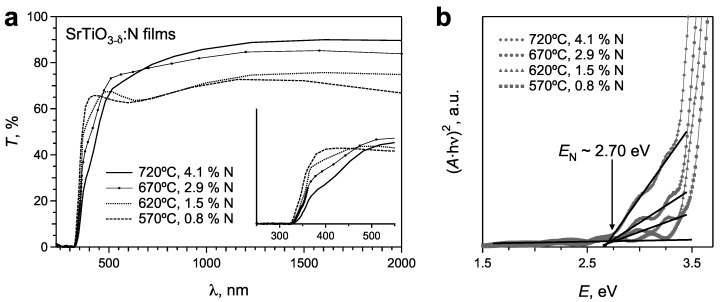
(**a**) Transmittance spectra of SrTiO_3_:N films deposited with the ammonia gas pulse at different substrate temperatures. The insert illustrates the increase of the absorption within the “N absorption shoulder” wavelength region with an increase of the nitrogen content. (**b**) The corresponding Tauc plots for these films. The increase of the nitrogen content to 4.1 at.% does not change the energy of N levels inside the band gap.

As mentioned above, the nitrogen content in SrTiO_3_:N films deposited with the ammonia gas pulse increases considerably with an increase of the substrate temperature (Figure 5). It is therefore possible to study the influence of a larger N content on the optical properties of the films. The transmission spectra of SrTiO_3_:N films deposited on LaAlO_3_ substrates with the ammonia gas pulse at different substrate temperatures are shown in Figure 8a.

The absorption shoulder at λ of 367‑460 nm increases with an increase of the nitrogen content in the films grown at higher temperatures due to the larger density of the N(2*p*) states inside the band gap. The Tauc plots confirm this observation and reveal no essential change of the energy of the nitrogen levels for different N contents in the films (Figure 8b). The films grown at higher substrate temperatures exhibit in addition to the enhanced optical absorption in the range of 367‑460 nm also a higher transmittance in the IR region, which can be attributed to the smaller Ti^3+^ content in the films. Both factors (i.e., increased optical absorption in the visible range and lower Ti^3+^ content) are favorable for possible photocatalytic applications. Hence, the most promising conditions for the deposition of SrTiO_3_:N films for photocatalytic applications are PRCLA with ammonia for the gas pulse and substrate temperatures above 700 °C.

## 4. Conclusions

We have shown that a one‑step preparation of textured N‑substituted SrTiO_3_ thin films by PRCLA on MgO and LaAlO_3_ substrates is possible. The nitrogen content in the films can be tailored by a selection of the nitridizing source and adjusting the deposition parameters, i.e., mainly the substrate temperature. Films grown with the N_2_ gas pulse reveal lower N contents of 0.99 ± 0.16 at.% as compared to films deposited with NH_3_ with an average N content of 2.31 ± 0.42 at.%. The N content in films deposited with the NH_3_ gas pulse increases from 0.76 ± 0.27 to 4.06 ± 0.39 at.% with increasing the substrate temperature (570 °C to 720 °C). The incorporation of nitrogen in SrTiO_3_ results in the formation of localized N(2*p*) states located above the top of the valence band in the energy diagram. This leads to an optical absorption of visible light at 370‑460 nm, which may be of potential interest for photo catalytic applications. The optical absorption at these wavelength increases with an increase of the nitrogen content in the films. The best conditions for the deposition of SrTiO_3_:N films with the highest N content and the smallest Ti^3+^ concentration are: PRCLA with an ammonia gas pulse and a substrate temperature above 700 °C.
